# Seasonal Trends in the Prevalence and Incidence of Viral Encephalitis in Korea (2015–2019)

**DOI:** 10.3390/jcm12052003

**Published:** 2023-03-02

**Authors:** Seon Jin Lee, Jeong Min Kim, Ha Rim Keum, Sang Won Kim, Hee Sun Baek, Jun Chul Byun, Yu Kyung Kim, Saeyoon Kim, Jae Min Lee

**Affiliations:** 1 Department of Medicine, College of Medicine, Yeungnam University, Daegu 42415, Republic of Korea; 2 Medical Research Center, College of Medicine, Yeungnam University, Daegu 42415, Republic of Korea; 3 Department of Pediatrics, College of Medicine, Yeungnam University, Daegu 42415, Republic of Korea; 4 Department of Pediatrics, School of Medicine, Keimyung University, Daegu 42601, Republic of Korea; 5 Department of Clinical Pathology, School of Medicine, Kyungpook National University, Daegu 41944, Republic of Korea

**Keywords:** encephalitis, respiratory syncytial virus, coronavirus, influenza virus, norovirus

## Abstract

Viral infections are a common cause of encephalitis. This study investigated the relationship between the incidence of encephalitis and that of respiratory and enteric viral infections in all age groups from 2015 to 2019, using the Health Insurance Review and Assessment (HIRA) Open Access Big Data Platform. We identified monthly incidence patterns and seasonal trends using the autoregressive integrated moving average (ARIMA). The Granger causality test was used to analyze correlations between encephalitis incidence and the positive detection rate (PDR) at 1-month intervals. A total of 42,775 patients were diagnosed with encephalitis during the study period. The incidence of encephalitis was highest in the winter (26.8%). The PDRs for respiratory syncytial virus (HRSV) and coronavirus (HCoV) were associated with the trend in encephalitis diagnosis in all age groups, with a 1-month lag period. In addition, an association with norovirus was observed in patients aged over 20 years, and with influenza virus (IFV) in patients aged over 60 years. This study found that HRSV, HCoV, IFV, and norovirus tended to precede encephalitis by 1 month. Further research is required to confirm the association between these viruses and encephalitis.

## 1. Introduction

Encephalitis is inflammation of the brain parenchyma that causes serious neurological dysfunction in both adults and children. There are numerous causes of encephalitis. Among them, the most common are viral infections such as herpes simplex virus (HSV) or autoimmune responses such as N-methyl D-aspartate receptor antibodies [1]. It presents with fever, headache, seizures, and an altered mental state. For the diagnosis of encephalitis, there must be altered consciousness persisting for longer than 24 h, evidence of central nervous system (CNS) inflammation, and at least two of the following characteristic clinical findings: (1) fever; (2) seizures or focal neurological findings attributable to the brain parenchyma; (3) CSF pleocytosis (more than four white blood cells per µL); (4) EEG findings suggestive of encephalitis; and (5) neuroimaging findings suggestive of encephalitis [2]. Encephalitis is generally more common in children, but the epidemiology of each type varies according to age, country, season, viral genetic mutations, and the patients’ immune status [3].

Studies regarding the association between encephalitis and common viruses in Korea are scarce. Thus, we aimed to analyze public health data provided by the Health Insurance Review and Assessment (HIRA) Open Access Big Data Platform and the Korea Disease Control and Prevention Agency (KDCA) to determine the relationship between the incidence of respiratory and gastrointestinal viral infections and encephalitis.

## 2. Materials and Methods

### 2.1. Study Population

We extracted data regarding encephalitis from the HIRA, which is a government-affiliated organization created to build an accurate claims review and quality assessment system for the National Health Insurance, with databases open to all academic investigators [4,5,6]. The claims data in the HIRA database include patient diagnosis, treatment, procedures, surgical history, and prescription drugs, and serve as a valuable resource for healthcare service research. We studied the HIRA data of patients with encephalitis (A85, A850, A851, A852, A858, A86, G04, G040, G041, G042, G048, G049, G05, G050, G051, G052, and G058), including a total of 42,775 incident encephalitis cases reported between 1 January 2015 and 31 December 2019.

### 2.2. Viral Surveillance Data

We used the data reported by the KDCA on viruses that cause acute respiratory infections and gastroenteritis, including data on more than 4000 respiratory and 2000 enteric specimens collected from 17 local environmental and health institutes and over 100 participating hospitals across Korea during each year of the study period. The causative pathogens were then identified using standardized diagnostic procedures in a central laboratory, in which the pathogen prevalence was surveyed weekly and analyzed based on genetic testing of samples from patients with influenza-like illness or acute diarrhea. The positive detection rate (PDR) data were collected from 2015 to 2019 by calculating the average monthly PDRs of seven respiratory viruses (adenovirus [HAdV], parainfluenza virus [HPIV], respiratory syncytial virus [HRSV], influenza virus [IFV], coronavirus [HCoV], rhinovirus [HRV], and bocavirus [HBoV]) and four acute diarrhea viruses (HAdV, rotavirus, norovirus, and astrovirus).

### 2.3. Statistical Analysis

For the incidence rate calculations, we used the 2015 Korean population data, reported by the Ministry of the Interior and Safety, as the denominator, with a total population of 51,529,338. We then constructed a model of variations in encephalitis diagnosis using the autoregressive integrated moving average (ARIMA) modeling approach, which assumes that the current observation is related to past observations over time as previously analyzed. The general multiplicative form of the ARIMA model was denoted as (p, d, q), where p, d, and q were the order values of the non-seasonal autoregressive, differencing, and moving-average parameters, respectively. Additionally, the autocorrelation function (ACF) was examined to identify the general form of the model to fit. Considering the ACF graphs, different ARIMA models were identified for model selection (Supplementary Figure S1), and the minimum Akaike information criterion model was chosen as the best-fit model (Supplementary Table S1). Moreover, the Granger approach was used to investigate the number of current values in the time series y that could be described as other values [7,8,9]. The data were analyzed using R software (R Foundation for Statistical Computing, Vienna, Austria), and significance was defined as *p* < 0.05.

The annual incidence was determined using the number of individuals with encephalitis as the numerator and the annual Korean population based on the HIRA database as the denominator, multiplied by the corresponding age population in 2015. The annual incidence rate of encephalitis was standardized to the 2015 population. Relative risk was calculated by dividing the overall incidence of each age group by the overall incidence of the 20–39 age group.

## 3. Results

### 3.1. Patient Characteristics

During the 5-year period, 42,775 patients were diagnosed with encephalitis (Table 1). Of these, 7710 (18.0%) were aged 0–9 years, 4224 (9.9%) were aged 10–19 years, 8890 (20.8%) were aged 20–39 years, 11,233 (26.3%) were aged 40–59 years, and 10,718 (25.1%) were aged 60 years and over. A total of 20,760 (48.5%) male and 22,015 (51.5%) female patients (M:F ratio = 1:1.06) were included in the study.

The incidence rate of encephalitis was highest in the 0–9-years age group (35.1/100,000 person-years). The encephalitis incidence rate in the 0–9-years age group was 2.79-fold higher than that in the 20–39-years age group. The incidence rate per year was lowest in 2015 (7.5/100,000 person-years) (Table 2). 

### 3.2. Encephalitis Trend Analysis

**Figure 1:**
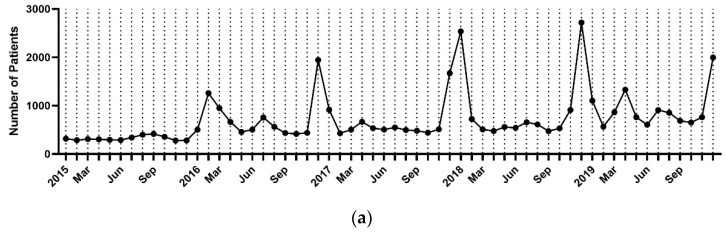

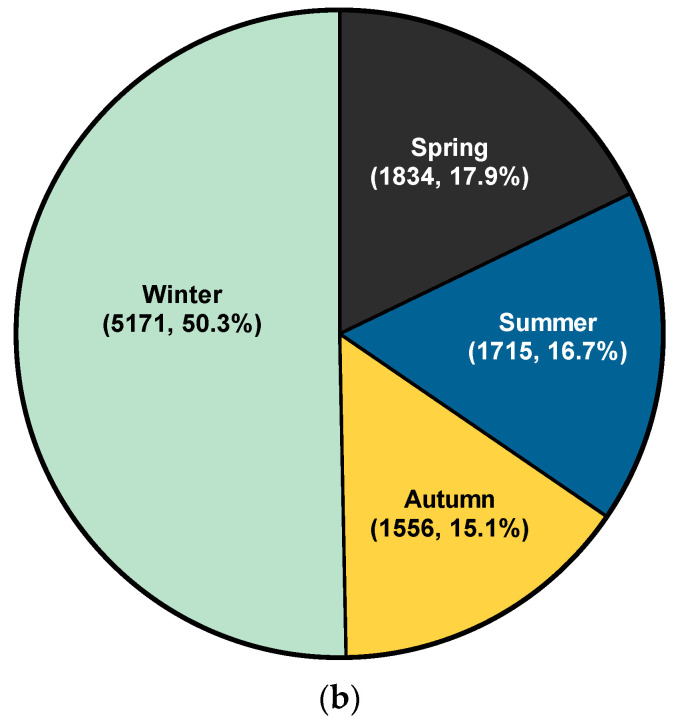
(**a**) Monthly trend analysis of encephalitis from 2015 to 2019. (**b**) Seasonal trend analysis of encephalitis incidence. Spring (March to May), summer (June to August), autumn (September to November), and winter (December to February). Cumulative incidence for 5 years, %.

Of the 42,775 patients of all ages, 3865 were diagnosed in 2015, 8883 in 2016, 7702 in 2017, 11,239 in 2018, and 11,086 in 2019 (Table 3). In 2015, the incidence rate was highest in September, and in 2016–2019, it was highest in December (Figure 1a). Overall, the cumulative number of cases per month over the 5-year study period was highest in December and lowest in October. The average number of cases per month was 540, and the average number of cases per year between 2015 and 2019 was 71,341. Additionally, encephalitis was most often diagnosed during winter (50.3%), followed by spring (17.8%), summer (16.7%), and autumn (15.1%) (Figure 1b).

**Figure 2:**
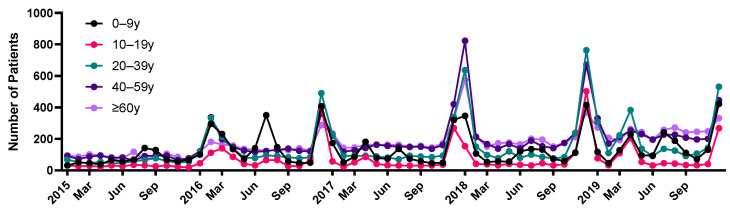
Monthly trend analysis of encephalitis according to age.

In addition, the seasonal trends in the incidence rate were similar in all age groups, increasing in the winter (Figure 2).

### 3.3. Viral Positive Detection Rates

The PDRs of most viruses showed seasonal variation (Supplementary Table S2, Figures S2 and S3). Specifically, the PDR of HAdV was highest from August to November; the PDR of HPIV was highest in May; the PDR of HBoV was highest in May and June; and the PDR of HRSV was highest in November. Furthermore, the PDR of HCoV and norovirus were highest from November to January; the PDR of HRV and enteric HAdV were highest in September; and the PDR of human metapneumovirus (HMPV), rotavirus, and astrovirus were highest in April, March, and January, respectively.

### 3.4. Causal Associations between Virus Prevalence and Encephalitis

The prevalence of viruses that cause encephalitis diagnosis might increase before the peak of encephalitis diagnosis. Thus, a Granger causality test was used to assess the association between the viral PDR and the number of encephalitis diagnoses reported 1 month later. The results are shown in Table 4.

**Figure 3:**
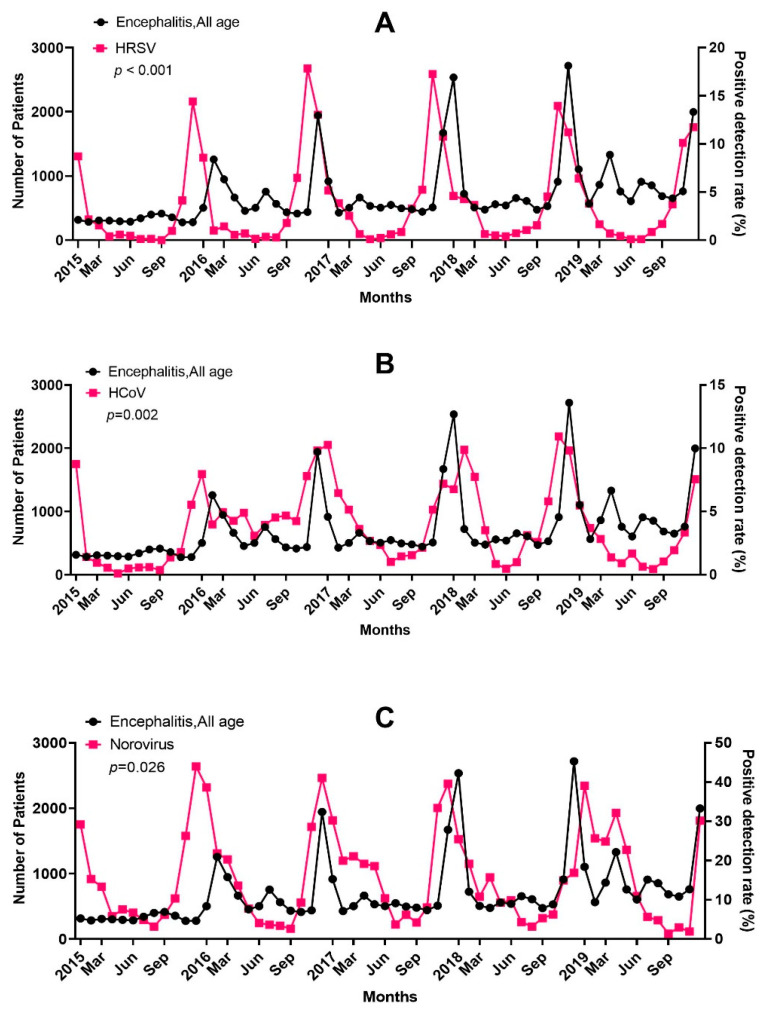
Relationship between the positive detection rate (PDR) of (**A**) respiratory syncytial virus (HRSV), (**B**) coronavirus (HCoV), and (**C**) norovirus and the incidence of encephalitis during the study period.

Among the seven respiratory viruses and four gastrointestinal viruses considered, the prevalence of certain viruses increased 1 month before the encephalitis incidence increased. The PDRs for HRSV (*p* < 0.001) and HCoV (*p =* 0.002) were associated with an increased incidence of encephalitis 1 month later in all age groups (Figure 3A,B). The PDR for norovirus (*p* = 0.026) was associated with an increased incidence of encephalitis 1 month later in patients aged 20 years and over (Figure 3C); the PDR for IFV (*p* = 0.041) was associated with an increased incidence of encephalitis 1 month later in patients aged 60 years and over.

## 4. Discussion

The causes of encephalitis are diverse and include infectious, autoimmune, and unknown causes. Infectious encephalitis is a serious disease that progresses with acute inflammation and can cause severe neurological sequelae or death. The most common cause is viral infection. Therefore, this study evaluated the incidence of encephalitis and its association with viral infections. We found that certain respiratory and gastroenteritis viral PDR were significantly associated with encephalitis incidence after 1 month.

The incidence of encephalitis is high globally. It is reported to be approximately 3 to 7 per 100,000 hospitalized patients [10,11,12]. Encephalitis can affect individuals of any age; however, it is more common in children. In the United States (US), the incidence was slightly higher in men before 1997 and has been higher in women since 1998 [13,14,15]. The incidence rate of encephalitis in the US was 7.3 per 100,000 person-years between 2000 and 2010, with a peak incidence rate in infants under 1 year (13.5 per 100,000 person-years) and the lowest incidence rate in youth aged 10 to 14 years (4.1 per 100,000 person-years) [16]. In France, the incidence rate of encephalitis was 2.6 people per 100,000 in 2007 [17]. In Western countries, the annual incidence of acute encephalitis is 7.4 per 100,000 population, with an annual incidence of 10.5 per 100,000 in children and 2.2 per 100,000 in adults [18]. The annual incidence in tropical countries is 6.34 per 100, 000 population.

The combined incidence of encephalitis in 204 countries increased from 1,284,160 cases in 1990 to 1,444,720 cases in 2019, an increase of 12.50% worldwide. However, during the last 30 years, the age-standardized incidence rate declined from 23.17 to 19.33 per 100,000 person-years [19]. Although population growth and advances in medicine led to these changes, encephalitis is still associated with high mortality rates and a high incidence of neurological sequelae.

In this study, the encephalitis incidence rate in Korea was 16.4 per 100,000 population between 2015 and 2019. Overall, a total of 42,775 patients were diagnosed with encephalitis, of whom 26.3% were aged 40–59 years, and 9.9% were aged 10–19 years. The male-to-female ratio was 0.94. The female predominance is consistent with the US data. The incidence rate was 35.1 per 100,000 population in the 0–9-years age group, and 19.7 per 100,000 population in the over 60 years age group.

Acute encephalitis constitutes a neurological emergency. The main cause of encephalitis is viral infection [20]. Viral encephalitis is caused by two distinct mechanisms [21]. Primary infectious encephalitis is caused by direct invasion of the CNS, followed by viral replication in the brain, whereas immune-mediated encephalitis results from CNS damage due to an abnormal immune response. Viruses such as arboviruses can invade the CNS through the blood–brain barrier, and HSV causes cytotoxicity to neurons [16].

Therefore, the peak season for encephalitis differs depending on the type of virus. In the US, the prevalence of arbovirus- and enterovirus-associated encephalitis is high in summer and fall [15]. In contrast, IFV, HAdV, and other seasonal respiratory viruses associated with encephalitis are most prevalent in winter. In this study, the prevalence of encephalitis in Korea was high in winter. However, the COVID-19 pandemic has changed the natural epidemic course of respiratory viruses such as HRSV and IFV [22,23,24,25,26,27]. Therefore, it is likely that the seasonal epidemiology of encephalitis has also changed since 2020 owing to changes in viral epidemiology.

Several studies have investigated the association between viruses (herpesvirus, enterovirus, arbovirus, mumps, measles, rabies, and Ebola) and encephalitis [28,29]. Respiratory viruses (HRSV, IFV, HCoV, and HMPV) have been identified as a cause of CNS pathology [30]. Although there are many probable causative viruses, no definitive answer has been obtained. We found that HRSV and HCoV prevalence were associated with encephalitis incidence in all age groups. In addition, IFV was significantly associated with the incidence of encephalitis in those aged 60 years or older.

HRSV appears to be infrequently associated with encephalitis. Saravanos et al. [31] conducted a systematic review of several acute neurological complications associated with acute HRSV infection in 155 children aged less than 15 years and confirmed 6.5% of children with HRSV infection had encephalitis/encephalopathy. HRSV mainly infects the epithelial cells of the lungs, but also affects the cells of the CNS. Several studies have found that HRSV can cause CNS complications such as encephalitis [27,30,32]. To the best of our knowledge, this is the first study to reveal an association between HRSV and encephalitis across all age groups in Korea.

Several studies have investigated the association between IFV and encephalitis. Muhammad et al. [33] analyzed 1244 children aged under 12 years with influenza A H1N1, and 13.6% of the patients who presented with influenza-related neurological manifestations were diagnosed with influenza-associated encephalitis (IAE). Morishima et al. [34] reported that 87.8% of 148 patients with IAE were infected with INV A, and the incidence of IAE was highest in children under 5 years of age. Acute IAE in adults is rare [35]. Underlying neurological diseases and young age are shown to be risk factors for influenza-associated neurological complications [36,37,38]. In contrast, there was a significant association between IFV and encephalitis incidence in adults over 60 years of age in our study. There are several possible reasons for the differences in the incidence of IAE between studies. First, there are no accepted diagnostic criteria for IAE in adults [35]. Second, older adults are susceptible to infection with IFV because of their weak immune systems and underlying diseases. Third, this may be related to the limitations of our study. Additionally, influenza vaccination is important for preventing influenza-related neurological complications. However, the effectiveness of influenza vaccination in preventing encephalitis is unclear [38,39]. Therefore, further studies are warranted.

Other respiratory viruses have also been reported to cause encephalitis. HMPV is thought to cause encephalitis in both children and adults [40,41,42]. CNS involvement by HAdV and HRV has been reported but is rare [43,44]. HBoV types 1, 2, and 3 have also been reported to be associated with encephalitis [45,46,47]. In our study, there were no significant differences between HAdV, HRV, HBoV, and HMPV prevalence and encephalitis incidence, which may be related to the limitations of our study.

In addition to respiratory viruses, some studies have investigated the association between gastrointestinal viruses and encephalitis. Among them, rotavirus is known to cause CNS complications [48]. There are three mechanisms by which rotavirus can cause CNS infection: a neural route from the site of infection to the brain through neural communication, direct infection of the CNS via the lymphatic system or immune mechanisms, and an indirect route that causes CNS effects via secondary messengers such as toxin or inflammation [49].

Rotavirus has been identified in half of the pediatric seizures associated with gastroenteritis in Japan [50]. Kasai et al. [51] found that rotavirus was the third most common cause of encephalitis, accounting for 45 of 1115 cases (4.0%) in 267 hospitals between 2014 and 2017. Takanashi et al. [52] confirmed that the clinical and magnetic resonance imaging characteristics of patients with rotavirus cerebellitis were similar. In addition, several cases of encephalitis associated with rotavirus have been reported [53,54,55,56,57,58]. However, in our study, there was no significant association between rotavirus prevalence and encephalitis incidence. Infants receive 2–3 doses of the rotavirus vaccine [59,60,61]. This may have contributed to the decrease in the incidence and severity of encephalitis in children.

Norovirus is a common cause of gastroenteritis, but reports of norovirus-associated encephalitis are rare [62,63,64,65]. However, studies conducted in Japan have shown that encephalitis associated with norovirus has a poor prognosis. Shima et al. [66] conducted a multicenter study in Japan on 29 children with norovirus-related encephalitis and confirmed that norovirus is one of the main causes of encephalitis, and that, in children with norovirus infection, early onset of neurological symptoms, an elevated serum creatinine level, and an abnormal blood glucose level are associated with poor prognosis. In our study, a significant correlation was found between the prevalence of norovirus and the incidence of encephalitis in those aged 20 years or older. The incidence of norovirus infection is lower than that of rotavirus infection; however, the prognosis of norovirus infection is worse. Therefore, further studies of the association between norovirus infection and encephalitis are needed [67,68].

According to an analysis of the US Healthcare Database from 2011 to 2014, enterovirus (58.4%) was the most common cause of encephalitis in infants and children under the age of 17 years [69]. During the same period, a study of 26,429 adult patients aged 18 years or older with encephalitis confirmed that enterovirus was the most common cause, accounting for 51.6% of all cases [70].

Chen et al. [71] confirmed that CNS infection with enterovirus causes encephalitis, which is especially common in young children, considering the potential causal link between the neuroimmune system and environmental neuroinvasion. There are three mechanisms whereby enterovirus may cause encephalitis. First, neurotropic viruses, including enterovirus, reach the CNS through the blood, pass through the blood–brain barrier, and directly act on the brain. Second, neurotropic viruses invade the CNS through immune cells infected with viruses circulating in the peripheral blood and reach the CNS through peripheral nerves [72,73,74,75,76].

Astroviruses rarely cause encephalitis and are not strongly associated with encephalitis [77,78,79]. There are only a few case reports, and no large-scale studies have been conducted. In our study, there was no association between astrovirus infection and the incidence of encephalitis.

Our study has several limitations. First, it was a retrospective study, which provides a lower level of evidence than prospective studies and may have had selection bias. Second, the data collected from the HIRA did not include patient clinical records. Third, the association between the prevalence of certain viruses and the incidence of encephalitis may not be causally related and may have been a chance finding. Moreover, the profile of patients tested for viral infections was different from that of patients diagnosed with encephalitis. Therefore, it was not possible to establish a direct relationship between viral infection and encephalitis since only a temporal relationship could be assessed. Therefore, the results may differ from those of previous studies. Although time-series analysis showed a trend between viral PDRs and encephalitis incidence rates using the Granger causality test, the results do not provide definitive evidence of a causal association between viral infection and encephalitis incidence. Because viral infections are common in winter, more epidemiological data are needed to link viral epidemics to the onset of encephalitis.

## 5. Conclusions

This study assessed the prevalence of common viral pathogens and their correlation with encephalitis. To our knowledge, this is the largest nationwide analysis of patients with encephalitis and its association with the PDRs of viruses in Korea. In Korea, increased incidence of HRSV, HCoV, IFV, and norovirus were found to precede the occurrence of encephalitis. It is possible that these viruses were the etiology of encephalitis. Prospective studies on these viruses and encephalitis are required to further elucidate these associations.

## Figures and Tables

**Table 1:** Characteristics of patients.

Variables		N (%)
Total number of patients		42,775 (100.0)
Age group		
	0–9.99 years	7710 (18.0)
	10~19.99 years	4224 (9.9)
	20~39.99 years	8890 (20.8)
	40–59.99 years	11,233 (26.3)
	≥60 years	10,718 (25.1)
Sex		
	Male	20,760 (48.5)
	Female	22,015 (51.5)
Location		
	Seoul	11,116 (26.0)
	Busan	3225 (7.5)
	Incheon	1927 (4.5)
	Daegu	1567 (3.7)
	Gwangju	2698 (6.3)
	Daejeon	1077 (2.5)
	Ulsan	582 (1.4)
	Gyeonggi	8656 (20.2)
	Gangwon	1930 (4.5)
	Chungbuk	706 (1.7)
	Chungnam	1492 (3.5)
	Jeonbuk	1594 (3.7)
	Jeonnam	911 (2.1)
	Gyeongbuk	1798 (4.2)
	Gyeongnam	2660 (6.2)
	Jeju	708 (1.7)
	Sejong	128 (0.3)
Insurance type		
	Medical insurance	40,839 (95.5)
	Medical aid	1918 (4.5)
	Free	18 (0.0)

**Table 2:** Incidence rate of encephalitis by age group.

Age Group (Years)	Annual Incidence * (Annual Incidence Rate) **					Overall Incidence Rate	Relative Risk ***
	2015	2016	2017	2018	2019		
0–9	818 (17.8)	2047 (44.5)	1375 (29.9)	1779 (38.7)	2067 (44.9)	35.1	2.79
10–19	331 (5.8)	1148 (20.1)	773 (13.5)	1230 (21.5)	1119 (19.6)	16.1	1.28
20–39	673 (4.7)	1894 (13.2)	1463 (10.2)	2554 (17.8)	2468 (17.2)	12.6	1.00
40–59	989 (5.8)	2039 (11.9)	2095 (12.2)	3226 (18.8)	2916 (17.0)	13.1	1.04
≥60	1054 (10.9)	1737 (18.0)	1919 (19.9)	2377 (24.6)	2429 (25.2)	19.7	1.56
Total	3865 (7.5)	8865 (17.2)	7625 (14.7)	11,166 (21.5)	10,999 (21.2)	16.4	1.30

* Total number of patients by year; ** All rates are per 100,000 population, directly age-adjusted to the 2015 population; *** Relative risk compared with that of the 20–39 years age group.

**Table 3:** Monthly numbers of newly diagnosed encephalitis patients in Korea.

Year	Jan	Feb	Mar	Apr	May	Jun	Jul	Aug	Sep	Oct	Nov	Dec	Total	Average
2015	316	285	308	304	294	288	341	399	415	357	278	280	3865	322
2016	503	1259	948	664	456	503	756	564	433	415	439	1943	8883	740
2017	917	428	503	665	533	506	549	496	481	442	510	1672	7702	642
2018	2535	723	508	476	559	540	656	610	473	530	911	2718	11,239	937
2019	1104	565	864	1330	760	606	909	854	685	651	761	1997	11,086	924
Total	5375	3260	3131	3439	2602	2443	3211	2923	2487	2395	2899	8610	42,775	3565
Average	946	513	488	512	447	466	550	491	391	362	403	917	71,341	540

**Table 4:** Causality of encephalitis incidence after 1 month with virus.

Age Group (Years)	HAdV	HPIV	HRSV	IFV	HCoV	HRV	HBoV	HMPV	Rotavirus	Norovirus	Adenovirus	Astrovirus
0–9	0.885	0.894	**<0.001**	0.521	**0.021**	0.447	0.556	0.377	0.965	0.112	0.589	0.668
10–19	0.89	0.345	**<0.001**	0.569	**0.004**	0.882	0.873	0.83	0.615	0.134	0.821	0.493
20–39	0.93	0.485	**<0.001**	0.572	**<0.001**	0.263	0.895	0.658	0.985	**0.046**	0.646	0.932
40–59	0.98	0.683	**<0.001**	0.139	**0.001**	0.072	0.789	0.646	0.662	**0.011**	0.545	0.898
≥60	0.542	0.66	**0.001**	**0.041**	**0.012**	0.097	0.463	0.497	0.596	**0.002**	0.512	0.913
total	0.938	0.63	**<0.001**	0.74	**0.002**	0.302	0.759	0.587	0.958	**0.026**	0.569	0.901

HAdV, human adenovirus; HPIV, human parainfluenza virus; HRSV, human respiratory syncytial virus; IFV, influenza virus; HCoV, human coronavirus; HRV, human rhinovirus; HBoV, human bocavirus; HMPV, human metapneumovirus. Statistically significant values less than 0.05 are written in bold.

## Data Availability

The data presented in this study are available on request from the corresponding authors.

## Supplementary Materials

The following supporting information can be downloaded at: https://www.mdpi.com/article/10.3390/jcm12052003/s1, Table S1: Parameters of ARIMA models for patients with encephalitis by age; Table S2: Positive detection rates of the virus during the study period; Figure S1: Residual ACF correlogram and 95% confidence limits for encephalitis; Figure S2. Positive detection rates of viruses during the study period; Figure S3. Positive detection rates of viruses during the study period (plot).
