# Supplementary material for: Seasonal Trends in the Prevalence and Incidence of Viral Encephalitis in Korea (2015–2019)
Source: J Clin Med. 2023 Mar 2;12(5):2003. doi: 10.3390/jcm12052003 (PMC10003849; doi:10.3390/jcm12052003)
Supplement: Supplementary file 1 [file jcm-12-02003-s001.zip › jcm-2250029-supplementary.pdf]

Table S1. Parameters of ARIMA models for encephalitis patients by age.

Parameters	0-9 years	10-19 years	20-39 years	40-59 years	≥60 years	Total
p autoregressive	0	0	0	0	0	0
d difference	0	0	1	1	1	1
q moving average	2	0	0	0	0	0
AIC	721.66	711.75	768.93	753.53	680.08	916.73

AIC, Akaike's Information Criterion.

Table S2. Positive detection rates of virus during study period.

Mon PDR(%)	Jan	Feb	Mar	Apr	May	Jun	Jul	Aug	Sep	Oct	Nov	Dec
						**HAdV**						
2015	5.04	2.15	2.83	4.70	4.83	5.63	3.32	3.18	2.30	6.98	9.03	8.78
2016	9.18	3.88	3.93	5.54	6.53	6.95	7.16	9.48	9.58	6.50	5.83	3.24
2017	3.72	2.53	2.43	2.74	6.05	4.73	2.96	5.18	4.33	3.78	4.73	2.60
2018	2.98	2.80	4.25	5.38	5.45	6.80	5.82	9.55	13.33	11.30	10.70	7.63
2019	6.68	5.05	4.85	4.43	7.84	10.40	7.52	12.88	10.20	9.44	9.75	8.83
						**HPIV**						
2015	0.92	0.55	0.78	6.30	17.65	11.75	7.04	6.28	8.93	4.72	9.53	4.08
2016	2.24	1.30	1.40	5.60	16.55	16.23	9.68	10.25	8.68	4.56	1.95	0.78
2017	1.58	1.70	2.98	8.38	19.43	12.83	9.68	8.95	4.60	3.04	3.33	2.68
2018	0.84	1.60	3.25	9.44	19.10	16.78	11.12	7.75	4.33	4.00	1.75	1.03
2019	1.06	1.10	2.33	3.48	13.56	20.35	15.74	9.70	7.58	3.98	2.55	1.65
						**HRSV**						
2015	8.70	2.15	1.55	0.40	0.58	0.45	0.12	0.13	0.00	0.96	4.13	14.40
2016	8.56	1.00	1.43	0.56	0.70	0.15	0.34	0.28	1.78	6.46	17.83	13.02
2017	5.16	3.83	2.53	0.64	0.10	0.23	0.60	0.85	3.30	5.24	17.25	10.73
2018	4.58	4.25	3.65	0.64	0.50	0.40	0.72	1.05	1.55	4.52	13.95	11.20
2019	6.40	3.80	1.65	0.70	0.44	0.10	0.10	0.85	1.68	3.72	10.10	11.73
						**IFV**						
2015	22.80	48.85	45.28	25.96	3.10	0.33	0.14	0.00	0.38	0.10	0.48	1.53
2016	15.24	47.55	36.95	26.60	5.00	0.70	0.22	0.28	0.25	0.40	0.80	35.50
2017	26.20	7.70	8.95	12.72	5.03	2.20	1.10	0.55	0.93	0.66	4.05	41.55
2018	58.50	35.63	10.55	3.46	2.80	1.08	0.50	0.30	0.58	1.78	11.15	35.35
2019	28.20	9.13	20.80	34.85	10.52	2.10	0.40	0.35	0.63	3.44	10.08	23.95
						**HCoV**						
2015	8.74	1.40	0.95	0.56	0.10	0.50	0.58	0.60	0.38	1.38	1.80	5.53
2016	7.96	3.98	4.95	4.26	4.90	3.08	3.94	4.53	4.68	4.24	7.80	9.82
2017	10.26	6.45	5.15	3.62	2.68	2.35	1.02	1.45	1.55	2.14	5.15	7.20
2018	6.76	9.88	7.73	3.52	0.85	0.48	1.00	3.13	2.60	5.80	10.93	9.83

2019	5.48	3.70	2.83	1.38	0.92	1.68	0.64	0.45	1.05	1.94	3.35	7.55
**HRV**												
2015	8.80	7.73	10.08	15.84	17.10	19.30	21.62	18.10	25.40	28.82	29.03	16.78
2016	8.52	3.78	12.28	15.44	19.55	16.38	19.62	19.60	18.40	24.56	20.68	8.02
2017	9.56	15.40	20.90	18.66	14.78	17.78	21.40	30.63	33.10	24.68	23.88	6.83
2018	2.52	5.95	20.63	28.34	21.53	20.18	21.14	12.70	24.63	22.08	15.70	9.78
2019	8.68	14.33	18.85	16.43	19.82	17.45	23.00	13.45	21.48	22.62	19.65	11.45
**HBoV**												
2015	0.58	0.70	0.68	5.18	9.05	8.18	2.46	0.18	0.00	0.56	0.23	0.55
2016	1.06	0.83	1.93	3.86	4.55	2.80	1.06	1.15	0.83	0.30	0.25	0.30
2017	0.58	1.00	1.35	4.66	9.33	3.98	0.82	0.30	0.25	0.18	0.20	0.58
2018	0.26	0.70	0.10	0.88	4.35	7.18	5.78	0.55	0.63	0.60	0.85	0.88
2019	0.52	0.58	1.40	1.78	4.88	11.78	8.22	2.00	2.05	1.16	1.20	1.10
**HMPV**												
2015	0.66	0.40	0.98	3.64	7.10	4.20	1.48	0.33	0.13	0.46	0.43	1.68
2016	3.56	4.20	8.05	12.64	8.53	2.83	0.66	1.20	0.98	0.42	1.05	1.14
2017	4.76	11.10	16.38	14.14	4.80	0.63	0.56	0.10	0.00	0.76	0.60	0.28
2018	0.86	2.95	8.60	19.12	15.33	6.00	2.82	0.73	0.48	0.08	0.18	0.15
2019	0.62	1.68	6.60	9.60	17.10	8.88	4.24	2.98	1.95	1.18	0.70	1.63
**Group A Rotavirus**												
2015	14.10	22.35	28.90	16.38	6.33	2.70	1.72	3.25	5.38	2.62	1.65	0.98
2016	5.66	16.45	21.08	13.28	6.03	3.73	2.30	4.20	3.43	1.86	1.68	2.76
2017	10.38	21.68	30.33	20.26	8.13	5.53	1.68	2.30	3.45	2.34	4.35	7.38
2018	14.52	19.53	16.88	10.10	4.38	4.55	1.84	1.98	0.85	1.90	3.13	4.10
2019	5.06	11.88	13.48	4.93	5.12	0.90	1.32	1.45	1.00	0.96	3.60	2.75
**Norovirus**												
2015	29.20	15.30	13.35	5.82	7.53	6.73	4.86	3.20	6.18	10.36	26.28	43.98
2016	38.62	21.85	20.33	13.64	7.80	4.08	3.66	3.40	2.65	9.32	28.58	41.06
2017	30.28	20.00	21.08	19.18	18.58	10.40	3.72	6.20	4.23	8.06	33.40	39.58
2018	25.46	19.20	10.78	15.74	9.30	9.90	4.34	3.20	5.35	6.28	14.95	16.90
2019	39.10	25.70	24.88	32.15	22.74	10.95	5.66	4.80	1.38	2.96	1.93	30.15
**Enteric Adenovirus**												
2015	1.68	3.03	1.55	1.70	2.30	1.05	0.28	0.00	0.60	2.22	0.55	1.80
2016	2.28	2.58	1.25	2.30	2.28	2.75	2.16	3.43	10.73	6.88	5.68	6.78
2017	2.10	2.23	0.55	2.86	2.90	3.05	2.78	3.30	3.50	5.04	3.43	1.13
2018	2.40	1.85	2.55	3.04	2.85	5.65	4.44	6.45	6.03	3.82	3.33	2.15
2019	0.00	0.85	2.08	2.25	2.00	2.18	1.26	4.08	1.43	1.00	0.80	1.28
**Astrovirus**												
2015	2.30	1.28	2.30	2.68	3.25	3.05	0.36	0.58	1.73	1.14	0.70	0.68
2016	0.94	1.18	2.48	3.20	3.40	4.30	2.52	2.28	3.43	2.82	2.05	0.86
2017	11.96	1.15	1.73	5.54	3.90	4.08	3.32	2.30	1.78	2.08	0.68	2.58

2018	0.94	1.73	0.00	1.82	3.05	2.50	2.74	5.18	5.63	3.98	2.73	2.33
2019	1.44	3.05	2.70	0.98	1.26	2.20	3.14	1.50	3.23	1.28	4.80	1.50

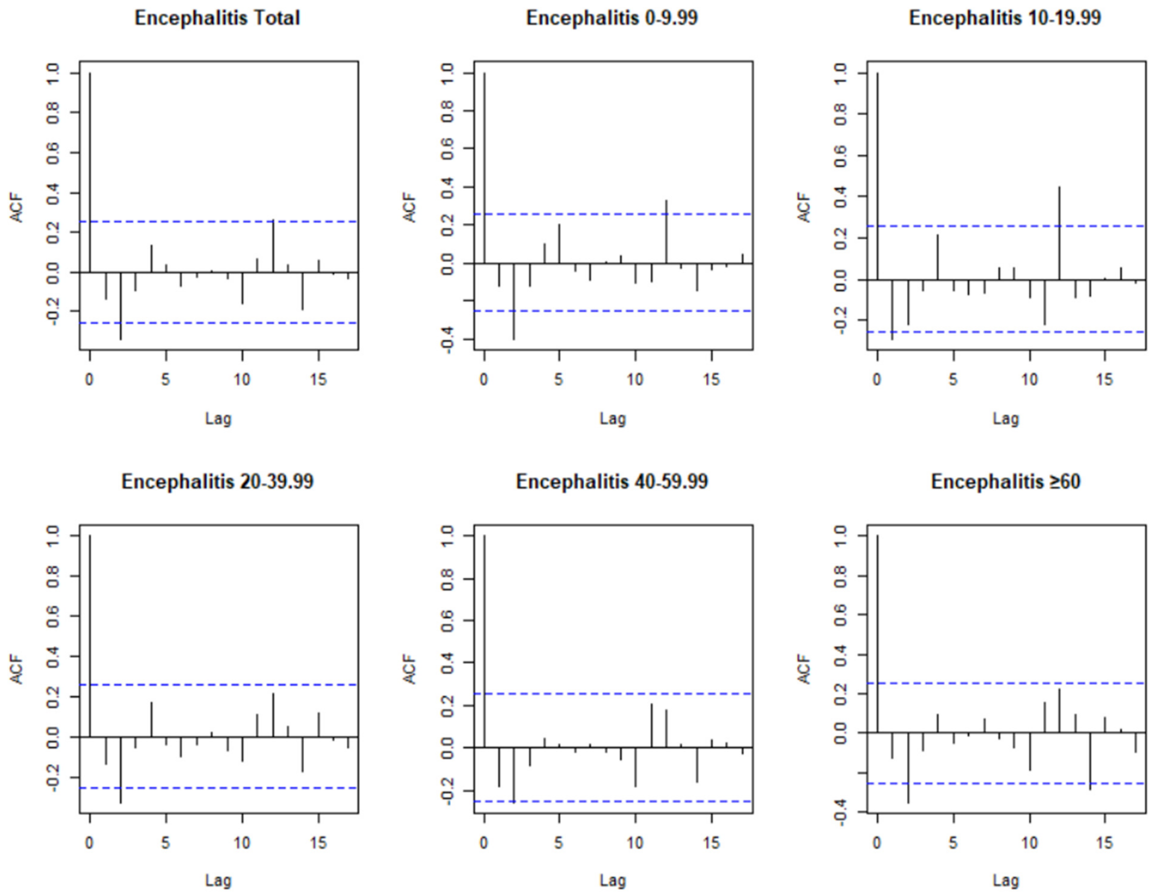

Figure S1. Residual ACF correlogram and 95% confidence limits for newly diagnosed encephalitis. ACF is a statistical technique to determine the degree of correlation between the values in a time series.

Mon / PDR(%)	Jan	Feb	Mar	Apr	May	Jun	Jul	Aug	Sep	Oct	Nov	Dec
**HAdV**												
2015	5.04	2.15	2.83	4.70	4.83	5.63	3.32	3.18	2.30	6.98	9.03	8.78
2016	9.18	3.88	3.93	5.54	6.53	6.95	7.16	9.48	9.58	6.50	5.83	3.24
2017	3.72	2.53	2.43	2.74	6.05	4.73	2.96	5.18	4.33	3.78	4.73	2.60
2018	2.98	2.80	4.25	5.38	5.45	6.80	5.82	9.55	13.33	11.30	10.70	7.63
2019	6.68	5.05	4.85	4.43	7.84	10.40	7.52	12.88	10.20	9.44	9.75	8.83
**HPIV**												
2015	0.92	0.55	0.78	6.30	17.65	11.75	7.04	6.28	8.93	4.72	9.53	4.08
2016	2.24	1.30	1.40	5.60	16.55	16.23	9.68	10.25	8.68	4.56	1.95	0.78
2017	1.58	1.70	2.98	8.38	19.43	12.83	9.68	8.95	4.60	3.04	3.33	2.68
2018	0.84	1.60	3.25	9.44	19.10	16.78	11.12	7.75	4.33	4.00	1.75	1.03
2019	1.06	1.10	2.33	3.48	13.56	20.35	15.74	9.70	7.58	3.98	2.55	1.65
**HRSV**												
2015	8.70	2.15	1.55	0.40	0.58	0.45	0.12	0.13	0.00	0.96	4.13	14.40
2016	8.56	1.00	1.43	0.56	0.70	0.15	0.34	0.28	1.78	6.46	17.83	13.02
2017	5.16	3.83	2.53	0.64	0.10	0.23	0.60	0.85	3.30	5.24	17.25	10.73
2018	4.58	4.25	3.65	0.64	0.50	0.40	0.72	1.05	1.55	4.52	13.95	11.20
2019	6.40	3.80	1.65	0.70	0.44	0.10	0.10	0.85	1.68	3.72	10.10	11.73
**IFV**												
2015	22.80	48.85	45.28	25.96	3.10	0.33	0.14	0.00	0.38	0.10	0.48	1.53
2016	15.24	47.55	36.95	26.60	5.00	0.70	0.22	0.28	0.25	0.40	0.80	35.50
2017	26.20	7.70	8.95	12.72	5.03	2.20	1.10	0.55	0.93	0.66	4.05	41.55
2018	58.50	35.63	10.55	3.46	2.80	1.08	0.50	0.30	0.58	1.78	11.15	35.35
2019	28.20	9.13	20.80	34.85	10.52	2.10	0.40	0.35	0.63	3.44	10.08	23.95
**HCoV**												
2015	8.74	1.40	0.95	0.56	0.10	0.50	0.58	0.60	0.38	1.38	1.80	5.53
2016	7.96	3.98	4.95	4.26	4.90	3.08	3.94	4.53	4.68	4.24	7.80	9.82
2017	10.26	6.45	5.15	3.62	2.68	2.35	1.02	1.45	1.55	2.14	5.15	7.20
2018	6.76	9.88	7.73	3.52	0.85	0.48	1.00	3.13	2.60	5.80	10.93	9.83
2019	5.48	3.70	2.83	1.38	0.92	1.68	0.64	0.45	1.05	1.94	3.35	7.55
**HRV**												
2015	8.80	7.73	10.08	15.84	17.10	19.30	21.62	18.10	25.40	28.82	29.03	16.78
2016	8.52	3.78	12.28	15.44	19.55	16.38	19.62	19.60	18.40	24.56	20.68	8.02
2017	9.56	15.40	20.90	18.66	14.78	17.78	21.40	30.63	33.10	24.68	23.88	6.83
2018	2.52	5.95	20.63	28.34	21.53	20.18	21.14	12.70	24.63	22.08	15.70	9.78
2019	8.68	14.33	18.85	16.43	19.82	17.45	23.00	13.45	21.48	22.62	19.65	11.45
**HBoV**												
2015	0.58	0.70	0.68	5.18	9.05	8.18	2.46	0.18	0.00	0.56	0.23	0.55
2016	1.06	0.83	1.93	3.86	4.55	2.80	1.06	1.15	0.83	0.30	0.25	0.30
2017	0.58	1.00	1.35	4.66	9.33	3.98	0.82	0.30	0.25	0.18	0.20	0.58
2018	0.26	0.70	0.10	0.88	4.35	7.18	5.78	0.55	0.63	0.60	0.85	0.88
2019	0.52	0.58	1.40	1.78	4.88	11.78	8.22	2.00	2.05	1.16	1.20	1.10
**HMPV**												
2015	0.66	0.40	0.98	3.64	7.10	4.20	1.48	0.33	0.13	0.46	0.43	1.68
2016	3.56	4.20	8.05	12.64	8.53	2.83	0.66	1.20	0.98	0.42	1.05	1.14
2017	4.76	11.10	16.38	14.14	4.80	0.63	0.56	0.10	0.00	0.76	0.60	0.28
2018	0.86	2.95	8.60	19.12	15.33	6.00	2.82	0.73	0.48	0.08	0.18	0.15
2019	0.62	1.68	6.60	9.60	17.10	8.88	4.24	2.98	1.95	1.18	0.70	1.63
**Group A Rotavirus**												
2015	14.10	22.35	28.90	16.38	6.33	2.70	1.72	3.25	5.38	2.62	1.65	0.98
2016	5.66	16.45	21.08	13.28	6.03	3.73	2.30	4.20	3.43	1.86	1.68	2.76
2017	10.38	21.68	30.33	20.26	8.13	5.53	1.68	2.30	3.45	2.34	4.35	7.38
2018	14.52	19.53	16.88	10.10	4.38	4.55	1.84	1.98	0.85	1.90	3.13	4.10
2019	5.06	11.88	13.48	4.93	5.12	0.90	1.32	1.45	1.00	0.96	3.60	2.75
**Norovirus**												
2015	29.20	15.30	13.35	5.82	7.53	6.73	4.86	3.20	6.18	10.36	26.28	43.98
2016	38.62	21.85	20.33	13.64	7.80	4.08	3.66	3.40	2.65	9.32	28.58	41.06
2017	30.28	20.00	21.08	19.18	18.58	10.40	3.72	6.20	4.23	8.06	33.40	39.58
2018	25.46	19.20	10.78	15.74	9.30	9.90	4.34	3.20	5.35	6.28	14.95	16.90
2019	39.10	25.70	24.88	32.15	22.74	10.95	5.66	4.80	1.38	2.96	1.93	30.15
**Enteric Adenovirus**												
2015	1.68	3.03	1.55	1.70	2.30	1.05	0.28	0.00	0.60	2.22	0.55	1.80
2016	2.28	2.58	1.25	2.30	2.28	2.75	2.16	3.43	10.73	6.88	5.68	6.78
2017	2.10	2.23	0.55	2.86	2.90	3.05	2.78	3.30	3.50	5.04	3.43	1.13
2018	2.40	1.85	2.55	3.04	2.85	5.65	4.44	6.45	6.03	3.82	3.33	2.15
2019	0.00	0.85	2.08	2.25	2.00	2.18	1.26	4.08	1.43	1.00	0.80	1.28
**Astrovirus**												
2015	2.30	1.28	2.30	2.68	3.25	3.05	0.36	0.58	1.73	1.14	0.70	0.68
2016	0.94	1.18	2.48	3.20	3.40	4.30	2.52	2.28	3.43	2.82	2.05	0.86
2017	11.96	1.15	1.73	5.54	3.90	4.08	3.32	2.30	1.78	2.08	0.68	2.58
2018	0.94	1.73	0.00	1.82	3.05	2.50	2.74	5.18	5.63	3.98	2.73	2.33
2019	1.44	3.05	2.70	0.98	1.26	2.20	3.14	1.50	3.23	1.28	4.80	1.50

Figure S2. Positive detection rates of virus during study period.

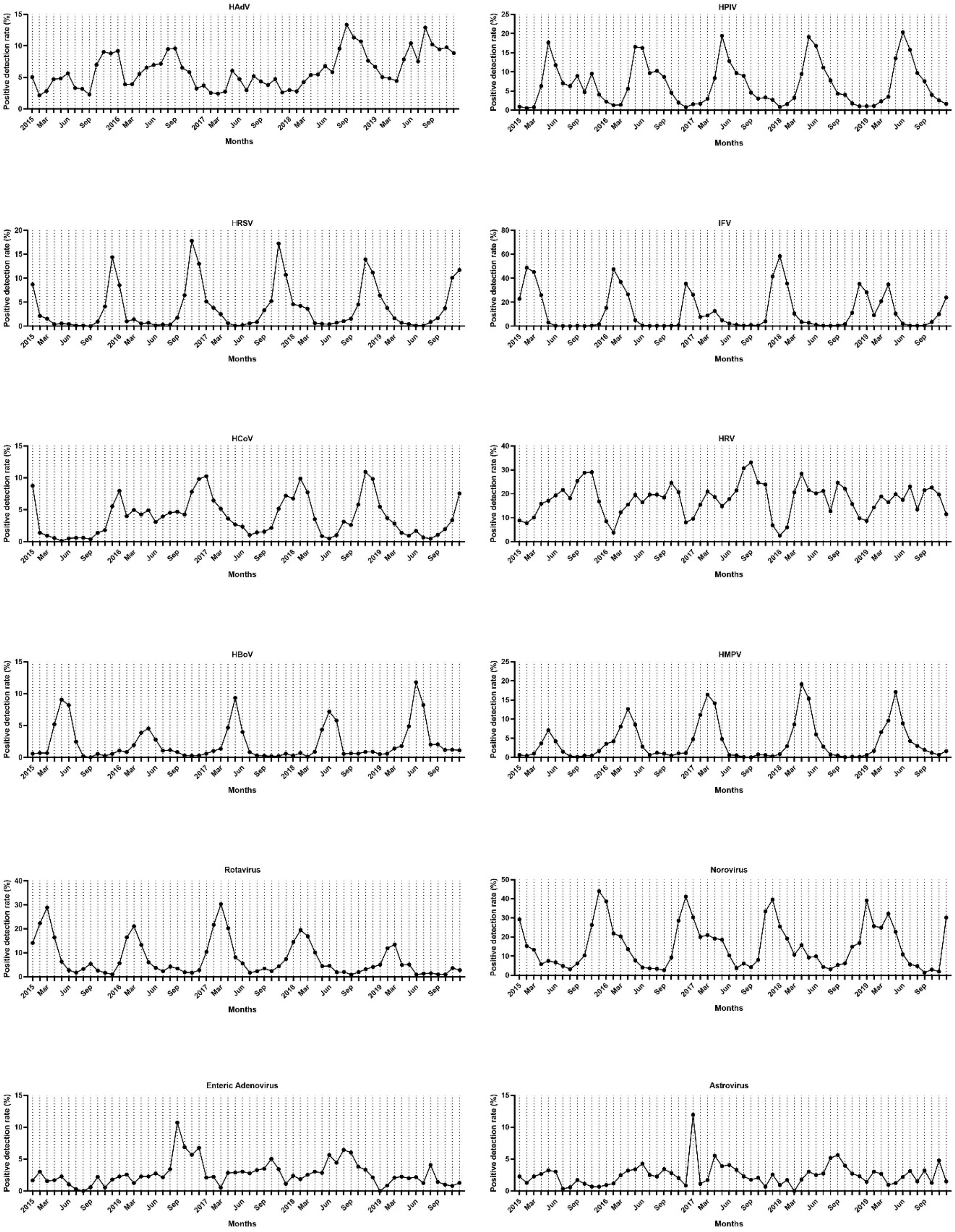

Figure S3. Positive detection rates of virus during study period (plot).
